# Costs related to immunopreventable diseases: Brazil and its geographic areas (immunopreventable diseases’ costs in Brazil)

**DOI:** 10.1186/s12913-021-07117-5

**Published:** 2021-10-27

**Authors:** Élide Sbardellotto M. da Costa, Adriano Hyeda, Eliane M. C. P. Maluf

**Affiliations:** 1 Department of Internal Medicine Post-graduation, Federal University of Paraná, General Carneiro Street, 181, Curitiba, Paraná Brazil; 2 Internal Medicine, Federal university of Paraná (UFPR), Health Management by Superior Institute of Management and Economy / Getúlio Vargas Foundation (ISAE/FGV), Curitiba, Brazil; 3 Pos-geraduation Program in Internal Medicine, Federal University of Paraná (UFPR), Curitiba, Brazil

**Keywords:** Hospital costs, Communicable diseases, Vaccines, Unified health system

## Abstract

**Introduction:**

The occurrence of the immunopreventable diseases in a population global reality.

**Objective:**

To discriminate the direct costs of the hospitalizations from the immunopreventable diseases in the Unified Health System (SUS), in Brazil and their areas, between 2008 and 2018.

**Methods:**

A population, observational, descriptive and retrospective study, with data from the information supplied by the DATASUS website, these data were collected during the 2019.

**Results:**

It was identified 457,479 hospitalizations in the period; with a total of 2,450,870 days of hospital stay, with total costs of R$389,243,264.85. Only the disease mumps presented a growing tendency in whole areas; the chickenpox was decreasing; the illness whooping cough, yellow fever and tetanus were stationary in this period.

**Conclusions:**

The costs related to immunopreventable diseases were relevant in all the Brazil areas, with tendencies different between the geographic areas and between the several illnesses analysed.

## Introduction

The World Health Organization (WHO) estimates that a fourth of the deaths in children under five years old are caused by preventable diseases [1–7]. According to the international literature [8–10], an important proportion of the health care is attributed to communicable diseases, one in six cases assisted by the primary health care, and about 128,000 hospitalizations (84% in public hospitals) were related to these conditions (data from 2010).

Therefore, it is a national public interest the rising of data by health care in this context of preventable diseases. Other countries already had made similar researches with very relevant data about the Immunizations Programs interfere positively in the reduction of the care costs and of rehabilitation, productivity increase, reduction of work absenteeism, and indirect social impact from these diseases [11, 12]. In this context, it is necessary an analysis of the Brazilian reality of the immunopreventable diseases, considering that the Immunization Program is a national reality [13], and considering the campaigns anti-vaccination and the fall of the vaccination coverages in all Brazilian geographic area (the national vaccination coverages don’t reach the minimum of 95% of coverage - Table 1) and in the whole world [8].

**Table 1:** Description of the immunization coverage, discriminated by each vaccine and by Brazilian geographic area, in the period from 2008 to 2021

Vaccine	Brazilian geographic area					
	North	Northeast	Southeast	South	Center-west	Total
Calmette-Guérin bacillus vaccine (BCG)	103.62	100.08	97.81	97.71	103.68	99.55
Hepatitis B vaccine (HBV) in children under 30 days	72.28	72.36	71.74	67.95	79.08	72.06
Rotavirus Humano G1P1 vaccine (VORH)	74.21	83.25	89.07	90.00	89.45	85.93
Meningococal C vaccine (MenC)	68.56	72.93	84.12	82.84	79.82	78.60
Hepatitis B vaccine (HBV)	84.64	90.18	93.30	93.51	93.31	91.49
5-in-1 vaccine (diphtheria, tetanus, whooping cough, Haemaphilus influenzae B, hepatitis B)	69.09	75.73	81.03	82.82	82.38	78.58
Pneumococcal 10 vaccine (Pnc10)	71.10	79.35	86.34	88.50	86.36	82.98
Poliomielitis 1, 2, 3 vaccine (VIP)	86.48	91.80	93.15	92.66	94.50	92.08
Poliomielitis 1,2,3 vaccine in children under 4 years	45.27	52.69	73.85	84.34	73.64	65.35
Yellow fever vaccine	80.29	39.64	40.37	56.10	83.75	50.10
Hepatitis A vaccine	58.06	64.09	69.39	72.75	70.84	67.24
Pneumococcal 10 vaccine third dose	71.03	79.46	83.34	85.88	85.17	81.40
Meningococcal C vaccine third dose	73.28	81.72	84.91	88.29	87.25	83.40
Poliomielitis 1,2,3 vaccine oral dose (VOP)	65.23	74.54	79.38	79.93	80.42	76.65
3-in-1 virus vaccine (MMR or SCR - measles, mumps, rubella) first dose	92.28	98.24	96.00	94.60	96.76	96.13
3-in-1 virus vaccine (MMR or SCR - measles, mumps, rubella) second dose	63.58	69.31	78.59	82.15	78.82	74.85
4-in-1 virus vaccine (SRC+VZ - measles, mumps, rubella, chickenpox)	55.71	39.95	46.04	69.60	68.20	50.46
3-in-1 vaccine (DTP - diphtheria, tetanus, whooping cough)	91.71	96.86	97.15	97.42	100.08	96.74
3-in-1 vaccine (DTP - diphtheria, tetanus, whooping cough) (in children between 4 and 6 years)	29.79	31.91	38.54	44.48	37.86	36.17
3-in-1 vaccine (DTP - diphtheria, tetanus, whooping cough) fourth dose	64.34	73.58	75.77	76.52	75.51	74.00
2-in-1 vaccine (dT - diphtheria, tetanus in adults) and 3-in-1 vaccine (dTpa - diphtheria, tetanus, whooping cough acelular) in pregnats	38.15	45.16	37.23	35.76	41.88	39.75
3-in-1 vaccine (dTpa - diphtheria, tetanus, whooping cough acelular) in pregnats	34.38	38.36	37.58	38.56	41.80	37.93
4-in-1 vaccine (DTP - diphtheria, tetanus, whooping cough) and Haemaphilus influenzae B	80.41	85.86	84.11	84.10	84.72	84.27
TOTAL	71.64	72.94	73.54	75.69	79.47	73.91

Source: Unique Health System Information (DATASUS - TABNET), data collected at March 2021

## Methods

In this manuscript, it was defined to analysis the data regarding the Brazil, discriminated by its geographical official country areas: North, Northeast, Center-west, Southeast and South.

It was accomplished a population study, observational, descriptive, retrospective, with multiple groups and temporary series, with aggregated public data, through information supplied by the public website of the System of information of the Department of the Unified Health System (DATASUS - http://www2.datasus.gov.br/DATASUS/index.php?area=02). The research methodology used by the website of DATASUS was established according to the available tools in the consultation system: through the following links: “Information of Health (TABNET)”, “Epidemiology and Morbidity”; “SUS Hospital morbidity (SIH/SUS) “; “General hospitalization place - starting from 2008”; “Brazil for Area and Federation Units”; Line = “Area / Federation Units”; Column = it hadn’t been “activated”, content = “Hospitalizations; Hospitalization approved authorizations (AIH); Total value; Value of the hospital services; Value of the professional services; AIH average value; days of hospital stay; Average days of hospital stay; Deaths; Mortality tax”; available period of January 2008 to December 2018; chapter of CID 10 = “infectious and parasitic diseases”; list of morbidities / CID 10 = “Neonatal tetanus and other tetanus; Diphtheria; Whooping cough; Yellow fever; Meningococcal disease; Measles; Rubella; Mumps; Human rabies virus; Chickenpox / Herpes Zoster; Acute hepatitis B” (these diseases had been chosen because they have preventable vaccines available in the Immunization Program from the Ministry of Health in Brazil, and their vaccination coverage – Table 1 – that consider just the first age year’s vaccine for the national coverage calc - available in the DATASUS site).

The analysed variables were the immunopreventable diseases above-mentioned, year, age groups, gender and economic variables. The partner-demographic data were tabulated and appraised for descriptive statistics (average and percentages, standard deviation SD, confidence interval CI), by the programs Excel® (Microsoft Corp., United States version 2007), Stata® (StataCorpLP, College Station, United States version 14.0), and Epi info 7®, by the research team. For the continuous variables (numeric), the analysis of lineal regression was used in the cases of verification of the correlations between the economic variables and immunopreventable disease. It was also analysed the temporary tendencies (Yt) of the economic variables correlated to the hospitalizations, the age groups and the gender, that was defined by the lineal regression equation Yt = b0 + b1t + et. In that expression, the parameter b0 corresponds to a constant, b1 corresponds to the inclination of the straight line and et is a random mistake, by the Prais-Winsten method. When the parameter Beta was positive, the temporary series was considered growing; when negative, it was considered decreasing; and stationary when there was no significant difference between its value and the zero. To measure the tax of variation of the straight line that adjusts the points of the temporary series the logarithmic transformation of base 10 of the coefficients it was accomplished (Y), because it contributes to the reduction of the heterogeneity of the variance for the residues of the analysis of lineal regression.

## Results

It was obtained data regarding 457,479 hospitalizations by immunopreventable disease, in the period from 2008 to 2018, in Brazil [minimum of 275 hospitalizations in the country per year; average of 41,589; maximum of 292,209; with standard deviation (SD) of +/− 87,366.40 with 95% confidence interval (95% CI) 253.16]. These hospitalizations in the period totaled 2,450,870 days of hospital stay in the period (minimum of 1486 days per year; maximum of 1,271,064 days; average of 222,806.36; SD +/− 384,716.64 with 95% CI 481.64). The total hospitalization costs in this period was R$389,243,264.85 (minimum of R$144,992.82 per year; maximum of R$216,785,771.98; average of R$35,385,751.35; SD +/− R$63,889,745.85 with 95% CI R$6347.00); being R$340,768,959.48 regarding the hospital services costs (87.55% of the total value); and R$48,471,910.33 regarding the total professional services costs during the hospitalizations (12.45% of the total value of the analysed hospitalizations) (Table 2). These data were updated at DATASUS system site in 2021.

**Table 2:** Description of the data regarding the hospitalizations, health care costs (total costs, hospital services and professional services), days of hospital stay and deaths, discriminated by immunopreventable disease, in Brazil, in the period from 2008 to 2018

Immunopreventable disease	Hospitalization	Total costs	Hospital services costs	Professional services costs	Total days of hospital stay
Mumps	4,097	R$ 961,818.65	R$ 808,993.78	R$ 152,749.07	18,067
Whooping cough	21,202	R$ 27,960,510.08	R$ 25,295,882.91	R$ 2,664,627.17	148,354
Diphtheria	1,354	R$ 3,140,701.53	R$ 2,761,629.70	R$ 378,883.02	11,970
Yellow fever	1,993	R$ 2,668,008.64	R$ 2,366,987.18	R$ 301,021.46	11,618
Influenza	292,209	R$ 216,785,771.98	R$ 188,719,311.10	R$ 28,065,572.91	1,271,064
Hepatitis B	15,415	R$ 13,038,202.36	R$ 11,466,699.57	R$ 1,571,401.42	146,890
Meningococcal disease	24,022	R$ 47,156,734.49	R$ 42,245,572.79	R$ 4,910,168.46	244,888
Rubella / German measles	275	R$ 144,992.82	R$ 122,983.92	R$ 22,008.90	1,486
Measles	1,566	R$ 497,605.16	R$ 419,208.96	R$ 78,396.20	7,707
Neonatal and accidental tetanus	2,208	R$ 11,547,254.34	R$ 10,146,762.21	R$ 1,400,492.13	36,581
Chickenpox/Herpes Zoster	93,138	R$ 65,341,664.80	R$ 56,414,927.36	R$ 8,926,589.59	552,245
TOTAL	**457,479**	**R$ 389,243,264.85**	**R$ 340,768,959.48**	**R$ 48,471,910.33**	**2,450,870**

Source: Unique Health System Information (DATASUS - TABNET). Period of the collected data from 2008 to 2018

Regarding the regional distribution of Brazil, it was observed that the different geographic areas of the country presented, in this analysed period, several behaviours in relation to each researched disease. The time series analysis (that was represented in the Table 3) demonstrated, in its majority, a stationary tendency of the illness costs in relation to the total of hospitalizations and to their total hospital costs. Some particularities were observed, as in the case of the disease mumps that presented growing tendency in all areas, with significant statistical (*p* < 0.05). The whooping cough came with stationary tendency (neither growing nor decreasing) in all areas in the period, the same case of yellow fever and tetanus (neonatal and accidental). Some sickness presented variations among the distributions in the different areas, like the case of diphtheria (that presented decreasing tendency in the Southeast and stationary in the other areas). The hospitalizations caused by influenza were decreasing in the Center-west, in the Southeast and in the South, and the total values of their hospitalizations were decreasing in the same States: Center-west and in the South. The total of hospitalization for hepatitis B declined in the North, South and Southeast, though, the total values of these hospitalizations were shown stationary in the period in all the country. The total of cases of meningococcal disease presented decreasing tendency in all areas in the period, except in the Northeast, though, the total values of these hospitalizations tended to decline only in the South area. The cases of hospitalizations for human rabies virus hospitalized had a decreasing tendency only in the Northeast, staying stationary during the period analysed in the other areas. In the case of the hospitalizations for German measles / rubella, both the total of internments and their total values tended to decrease only in the Southeast area, being shown a stationary tendency in the other areas of the country. The sickness measles came in very several ways of the others: the amount of hospitalizations came registered in system in a decreasing way in the area Center-west and shown a stationary tendency in the other areas; and the values associated its hospitalizations came growing in the Northeast (with *p*-value 0.009 and CI95% between 0.0264 and 0.136), decreasing in the North and in the other areas a stationary tendency. The sickness Chickenpox / Herpes Zoster showed a decreasing tendency of the total of hospitalizations in all the areas of the country in the period analysed (with statistical significance), though the total values of the hospitalizations came growing in the Northeast (with *p*-value 0.000 and CI95% between 0.015 and 0.028), decreasing in the North area (*p*-value 0.010 and CI95% between − 0.095 and − 0.017) and in the Southeast (p-value 0.032 with CI95% between − 0.048 and − 0.002).

**Table 3:** Tendency of the time series analysis from the data regarding the hospitalizations and health care costs (total costs, hospital services and professional services), discriminated by immunopreventable disease and Brazil geographic areas, in the period from 2008 to 2018

Immunopreventable disease by country areas	Hospitalizations					Total costs				
	Beta	P	CI95%		Tendency	Beta	P	CI95%		Tendency
**Mumps (2008-2018)**										
**BRAZIL**	0.052	0.001	0.026	0.0776	Growing	0.067	0.000	0.046	0.088	Growing
CENTER-WEST	0.043	0.023	0.007	0.079	Growing	0.067	0.006	0.025	0.110	Growing
NORTHEAST	0.058	0.000	0.035	0.079	Growing	0.060	0.004	0.024	0.097	Growing
NORTH	0.090	0.000	0.061	0.119	Growing	0.105	0.000	0.069	0.141	Growing
SOUTHEAST	0.041	0.004	0.017	0.066	Growing	0.063	0.000	0.049	0.076	Growing
SOUTH	0.061	0.007	0.021	0.100	Growing	0.065	0.001	0.032	0.099	Growing
**Whooping cough (2008-2018)**										
**BRAZIL**	0.026	0.549	-0.070	0.124	Stationary	0.033	0.468	-0.065	0.132	Stationary
CENTER-WEST	0.001	0.981	-0.107	0.109	Stationary	0.005	0.920	-0.106	0.116	Stationary
NORTHEAST	0.061	0.229	-0.046	0.168	Stationary	0.070	0.209	-0.047	0.188	Stationary
NORTH	0.038	0.378	-0.054	0.130	Stationary	0.041	0.297	-0.042	0.124	Stationary
SOUTHEAST	0.022	0.610	-0.075	0.121	Stationary	0.030	0.506	-0.069	0.130	Stationary
SOUTH	0.009	0.815	-0.083	0.103	Stationary	0.015	0.703	-0.074	0.105	Stationary
**Diphtheria (2008-2018)**										
**BRAZIL**	-0.029	0.018	-0.052	-0.0064	Decreasing	-0.024	0.235	-0.066	0.018	Stationary
CENTER-WEST	-0.072	0.058	-0.147	0.003	Stationary	-0.057	0.285	-0.170	0.056	Stationary
NORTHEAST	-0.005	0.806	-0.054	0.043	Stationary	0.007	0.844	-0.078	0.093	Stationary
NORTH	-0.054	0.180	-0.139	0.030	Stationary	-0.086	0.187	-0.223	0.050	Stationary
SOUTHEAST	-0.039	0.001	-0.056	-0.021	Decreasing	-0.042	0.001	-0.064	-0.021	Decreasing
SOUTH	-0.002	0.898	-0.052	0.046	Stationary	0.004	0.915	-0.081	0.090	Stationary
**Meningococcal disease (2008-2018)**										
**BRAZIL**	-0.043	0.011	-0.074	-0.012	Decreasing	-0.028	0.124	-0.065	0.009	Stationary
CENTER-WEST	-0.033	0.010	-0.056	-0.01	Decreasing	-0.029	0.094	-0.066	0.006	Stationary
NORTHEAST	-0.030	0.096	-0.068	0.006	Stationary	-0.006	0.779	-0.060	0.046	Stationary
NORTH	-0.018	0.007	-0.029	-0.006	Decreasing	-0.016	0.176	-0.041	0.009	Stationary
SOUTHEAST	-0.055	0.012	-0.095	-0.015	Decreasing	-0.039	0.072	-0.083	0.004	Stationary
SOUTH	-0.041	0.002	-0.062	-0.020	Decreasing	-0.029	0.004	-0.047	-0.012	Decreasing
**Yellow fever (2008-2018)**										
**BRAZIL**	0.123	0.241	-0.099	0.346	Stationary	0.182	0.206	-0.120	0.484	Stationary
CENTER-WEST	-0.027	0.258	-0.078	0.023	Stationary	-0.131	0.564	-0.629	0.366	Stationary
NORTHEAST	0.031	0.467	-0.061	0.123	Stationary	0.010	0.840	-0.100	0.120	Stationary
NORTH	-0.073	0.051	-0.147	0.000	Stationary	-0.025	0.569	-0.122	0.071	Stationary
SOUTHEAST	0.168	0.183	-0.096	0.433	Stationary	0.258	0.120	-0.082	0.599	Stationary
SOUTH	0.0008	0.985	-0.102	0.104	Stationary	0.0008	0.989	-0.134	0.135	Stationary
**Influenza (2008-2018)**										
**BRAZIL**	-0.023	0.001	-0.034	-0.013	Decreasing	-0.019	0.014	-0.033	-0.0052	Decreasing
CENTER-WEST	-0.077	0.002	-0.116	-0.037	Decreasing	-0.065	0.007	-0.106	-0.023	Decreasing
NORTHEAST	-0.011	0.425	-0.043	0.020	Stationary	-0.004	0.801	-0.047	0.038	Stationary
NORTH	-0.005	0.523	-0.026	0.014	Stationary	-0.003	0.778	-0.027	0.021	Stationary
SOUTHEAST	-0.034	0.037	-0.066	-0.002	Decreasing	-0.026	0.124	-0.060	0.008	Stationary
SOUTH	-0.026	0.014	-0.045	-0.006	Decreasing	-0.028	0.028	-0.053	-0.003	Decreasing
**Hepatitis B (2008-2018)**										
**BRAZIL**	-0.041	0.006	-0.068	-0.014	Decreasing	-0.012	0.365	-0.043	0.017	Stationary
CENTER-WEST	-0.026	0.078	-0.057	0.0037	Stationary	0.011	0.445	-0.021	0.045	Stationary
NORTHEAST	-0.028	0.084	-0.061	0.0046	Stationary	-0.008	0.575	-0.041	0.024	Stationary
NORTH	-0.055	0.038	-0.106	-0.0037	Decreasing	-0.035	0.112	-0.080	0.010	Stationary
SOUTHEAST	-0.039	0.024	-0.072	-0.006	Decreasing	-0.009	0.658	-0.057	0.038	Stationary
SOUTH	-0.061	0.005	-0.099	-0.024	Decreasing	-0.038	0.054	-0.077	0.0008	Stationary
**Human rabies virus (2008-2018)**										
**BRAZIL**	-0.056	0.036	-0.107	-0.004	Stationary	-0.008	0.738	-0.066	0.049	Stationary
CENTER-WEST	-0.064	0.055	-0.131	0.0018	Stationary	-0.200	0.187	-0.517	0.116	Stationary
NORTHEAST	-0.085	0.010	-0.145	-0.025	Decreasing	-0.057	0.120	-0.133	0.018	Stationary
NORTH	0.061	0.085	-0.010	0.134	Stationary	0.246	0.177	-0.133	0.626	Stationary
SOUTHEAST	-0.067	0.332	-0.215	0.081	Stationary	-0.036	0.768	-0.305	0.233	Stationary
SOUTH	-0.077	0.228	-0.212	0.057	Stationary	-0.102	0.516	-0.445	0.2400	Stationary
**Rubella / German measles (2008-2018)**										
**BRAZIL**	-0.056	0.002	-0.086	-0.025	Decreasing	-0.049	0.217	-0.133	0.034	Stationary
CENTER-WEST	-0.052	0.064	-0.109	0.0036	Stationary	-0.207	0.031	-0.391	-0.024	Decreasing
NORTHEAST	-0.055	0.062	-0.114	0.0035	Stationary	-0.050	0.051	-0.100	0.0003	Stationary
NORTH	-0.034	0.126	-0.080	0.0117	Stationary	-0.164	0.106	-0.372	0.043	Stationary
SOUTHEAST	-0.069	0.003	-0.108	-0.029	Decreasing	-0.047	0.036	-0.091	-0.003	Decreasing
SOUTH	-0.031	0.188	-0.081	0.018	Stationary	-0.029	0.296	-0.088	0.030	Stationary
**Measles (2008-2018)**										
**BRAZIL**	0.035	0.342	-0.044	0.115	Stationary	0.039	0.126	-0.013	0.092	Stationary
CENTER-WEST	-0.122	0.011	-0.208	-0.036	Decreasing	-0.132	0.270	-0.388	0.122	Stationary
NORTHEAST	0.050	0.067	-0.004	0.104	Stationary	0.081	0.009	0.0264	0.136	Growing
NORTH	0.020	0.515	-0.049	0.090	Stationary	-0.093	0.004	-0.146	-0.040	Decreasing
SOUTHEAST	-0.028	0.364	-0.096	0.038	Stationary	-0.034	0.171	-0.086	0.017	Stationary
SOUTH	-0.061	0.289	-0.185	0.062	Stationary	-0.063	0.369	-0.215	0.088	Stationary
**Neonatal and accidental tetanus (2008-2018)**										
**BRAZIL**	-0.003	0.224	-0.009	0.002	Stationary	0.013	0.079	-0.0018	0.028	Stationary
CENTER-WEST	0.0007	0.941	-0.019	0.021	Stationary	-0.008	0.697	-0.053	0.037	Stationary
NORTHEAST	-0.006	0.599	-0.033	0.020	Stationary	0.014	0.127	-0.0051	0.035	Stationary
NORTH	-0.008	0.446	-0.031	0.015	Stationary	0.031	0.225	-0.022	0.085	Stationary
SOUTHEAST	0.006	0.444	-0.012	0.025	Stationary	0.014	0.344	-0.018	0.049	Stationary
SOUTH	-0.003	0.765	-0.021	0.017	Stationary	0.009	0.102	-0.002	0.022	Stationary
**Chickenpox/Herpes Zoster (2008-2018)**										
**BRAZIL**	-0.037	0.000	-0.045	-0.029	Decreasing	-0.021	0.012	-0.036	-0.006	Decreasing
CENTER-WEST	-0.043	0.007	-0.071	-0.015	Decreasing	-0.030	0.107	-0.068	0.007	Stationary
NORTHEAST	-0.011	0.016	-0.021	-0.002	Decreasing	0.022	0.000	0.015	0.028	Growing
NORTH	-0.032	0.034	-0.061	-0.002	Decreasing	-0.056	0.010	-0.095	-0.017	Decreasing
SOUTHEAST	-0.040	0.000	-0.049	-0.031	Decreasing	-0.025	0.032	-0.048	-0.002	Decreasing
SOUTH	-0.049	0.008	-0.081	-0.016	Decreasing	-0.038	0.103	-0.087	0.009	Stationary

Source: Unique Health System Information (DATASUS - TABNET). Period of the collected data from 2008 to 2018

## Discussion

The immunopreventable diseases answer for half of all the deaths in countries with smaller development index: 90% of these deaths attributed to diarrheal diseases, breathing diseases, AIDS, tuberculosis, malaria and measles [12, 14–16], and many of them could be preventable through the vaccines. Based on the data collected in this research, still today citizens get sick and are hospitalized by immunopreventable diseases. As we observed in in this study, there were 457,479 hospitalizations in the period from 2008 to 2018, in Brazil with a budget impact of R$389,243,264.85. According to the Ministry of Health data (https://saude.gov.br/noticias/agencia-saude/45877-secretary-national-of-surveillance-in-greet-speech-on-supply-and-budget-for-vaccine-haul-of-investment-for-to-area), the budget destined to PNI corresponds to 53% of the general budget of the Health Surveillance Secretariat. This budget destined for purchase vaccines and inputs related to immunization totalized R$ 45.3 billion, according to the Annual Budget Bill (PLOA) of 2020. Considering the total budget of SUS R$147,43 billion for the health care in 2019, according to data of the Brazilian Office of Comptroller General(CGU) (http://www.portaltransparencia.gov.br/funcoes/10-saude?ano=2019), the expenses with immunopreventable disease hospitalizations are inside the total of R$114.18 billion of expenses executed for the area of the health (for hospital and outpatient care). In this context, analysing the direct costs with hospitalizations related to the immunopreventable diseases in SUS, it was observed the reflex of the actions of the public politics of health indirectly, mainly in the country, independent of the regional differences that knowingly exist. If was observed the total expenses here lifted, it is possible saving more than R$389 million avoiding diseases that can be forewarned for measures effective and thoroughly accessible for every Brazilian population = the vaccines.

Another observed interesting data is the fact that the hospitalizations for the immunopreventable disease didn’t decline in uniform way in the different country’s areas, and some illness until presented growing tendencies (as mumps, for instance). This situation should be analysed with very carefully, because it raises the existence of regional differences among health care quality, services structures installed, social inequalities that impact in the population’s health, and differences in the regional public politics of facing each immunopreventable disease (despite the national politics of prevention, promotion, diagnosis and treatment of each illness) [17–24].

Nowadays, the financial resources are being destined to the hospitalizations of Immunopreventable diseases can be forewarned with effective measures as the vaccines, if the population was appropriately vaccinated, these resources could be used for other situations, so many needs of public health. This manuscript brings an opportunity for improvement that is the importance of employing awareness public and private campaigns for the importance of increase the vaccination coverage in all geographic areas (Table 1), regarding the regional differences (differences in vaccination’s culture, health services access, health budget, health services territorial distribution, and many differences in the population reality that differ between the Brazilian geographic area).

All studies based on public secondary databases have the limitation, already known, of under-reporting and under-reporting of the analysed system itself, because these are dependent on the databases being fed by the employees responsible for the system. In the case of the SUS, these data are feeders in a decentralized manner and regionalized by States and Municipalities. However, despite the notorious underutilization of the system, these are the official data that are used for the development of public health policies in Brazil.

## Data Availability

The data that support the findings of this study are openly available in the information system website of the Department of the Unified Health System (DATASUS - http://www2.datasus.gov.br/DATASUS/index.php?area=02).
